# Remembering activism: Means and ends

**DOI:** 10.1177/17506980241262390

**Published:** 2024-10-09

**Authors:** Samuel Merrill, Ann Rigney

**Affiliations:** Umeå University, Sweden; Utrecht University, The Netherlands

**Keywords:** activist memory work, mediation, memory-activism nexus, mobilisation, protest, social movements

## Abstract

This editorial introduces the 12 articles collected in this special issue on *Remembering Activism: Explorations in the memory-activism nexu*s. It frames the articles within current debates in the field of memory studies and social movement studies on the entanglements between memory work, on the one hand, and activism directed towards social transformation, on the other. In particular, it highlights the ways in which the memory of earlier activism is mobilised within later movements; in the process, it also identifies various forms of activist memory work where remembrance is an integral part of the activist repertoire and one of the means used to achieve political ends.

The intersection between memory and activism has become a vital and still growing area of research within both memory studies and social movement studies. Titles such as *Negotiating Memories of Protest in Europe* (Hajek, 2013), *Cultural Memories of Non-Violent Protest* (Reading and Katriel, 2015), *Social Movements, Memory and Media* (Zamponi, 2018), *Social Movements, Cultural Memory and Digital Media* (Merrill et al., 2020) and *Remembering Social Movements: Activism and Memory* (Berger et al., 2021) testify to a cross-disciplinary acceleration of interest in the ways in which activists within and beyond social movements both call on and produce collective memory. This is just a sample from the rich harvest of monographs and edited collections, never mind research articles, that have appeared in the last decade with varying combinations of activism, social movements, protest and memory in their titles (see also Berger and Koller, 2024; Chidgey, 2018; Chidgey and Garde-Hansen, 2024; Daphi, 2017; Della Porta et al., 2018; Gutman, 2017; see also the review section of this special issue).

Within the field of social movement studies, the consideration of memory has arguably figured – for some time – as part of a subfield concerned with the role of culture in political mobilisation (see Baumgarten et al., 2014). Within the field of memory studies, however, the consideration of activism has recently gained a more central position and has even led to some scholars announcing an ‘activist turn in memory studies’ (Chidgey, 2023; Gutman and Wüstenberg, 2023). While the idea of an activist turn is not unproblematic (see Chidgey in this special issue), not least because it obfuscates the complex genealogy of current research debates and agendas, recent studies of the intersection between memory and activism have been opening up exciting new perspectives on the societal relevance of memory more broadly. However, some critical reflection on the assumptions underlying these studies is also needed at this juncture.

The apparent convergence of memory and activism, in fact, disguises differences in the origins of particular concepts and approaches. For instance, studies of ‘memory activism’, as pioneered by Yifat Gutman (2017) and Jenny Wüstenberg (2017), are closely aligned with traditions of research in transitional justice and in the politics and ethics of dealing with violent pasts. Indeed, the concept of memory activism, which has been used chiefly to understand activism directed towards mnemonic change, has proved very productive in bringing into visibility grassroots campaigns to change hegemonic narratives and bring silenced voices into circulation. Alternatively, other scholars have turned to the interaction between memory and activism with the aim of actually displacing legacies of violence as the central focus of research by, for example, highlighting instead the memory of non-violent struggles (Katriel, 2016; Reading and Katriel, 2015) and, linked to this, by calling for a more future-oriented memory studies that would link memory to hope and the possibility of social transformation rather than to trauma (Rigney, 2018). The recent convergence of these and other approaches has yielded a certain tension and sometimes friction between, on one hand, studies of grassroots attempts to change memory and, on the other hand, studies of the role of memory in grassroots attempts to change the world. Hence our concern here with the issue of ‘means’ and ‘ends’.

The term ‘digital activism’ offers a useful starting point for explaining this issue. In this particular collocation, the ‘digital’ refers to the means of activism rather than its ends. In ‘climate activism’, in contrast, ‘climate’ refers to the ends for which activists are mobilising. Taking into account such discriminations, we emphasise here the distinction between activism where mnemonic change is the goal, and activism directed towards social and political change in which memory is primarily a means rather than an end. This distinction needs emphasising since the term ‘memory activism’ has sometimes been taken up as a catchall term, encouraged by an ambiguous expansion of its original definition to encompass not just explicitly mnemonically oriented activism but any activism that uses memory as a primary resource in the pursuit of wider social or political causes (see Gutman and Wüstenberg, 2021, 2023; see also Merrill’s review in this issue). These conceptual tensions and debates reflect the teething problems associated with advancing a new research agenda across different interdisciplinary fields and, for that reason, they are to be embraced as an opportunity for further discussion and exploration.

This special issue on Remembering Activism seizes that opportunity. As its subtitle ‘Explorations in the memory-activism nexus’ suggests, the collection takes as its starting point the idea that the relationship between memory and activism entails a complex and dynamic set of relations rather than being a single phenomenon. Building on the concept of a ‘memory-activism nexus’ (Rigney, 2018), it sets out to explore further the empirical cases that are opened up by that term and to reflect further on their theoretical implications. The concept of the memory-activism nexus was first introduced to capture the primary ways in which memory and activism come to interact. It was accordingly originally defined in terms of the interplay between (1) *memory activism* – how activists struggle to produce cultural memory and steer future remembrance; (2) the *memory of activism* – how earlier activism is culturally recollected; and (3) *memory in activism* – how memory informs contemporary activism (see also Rigney, 2021).

The memory-activism nexus as a term is not, of course, without precedents: Reading and Katriel (2015) already referred in passing to the ‘under-explored nexus of memory-work and nonviolent activism’ (p. 4). Nor is it without alternatives, most notably the concept of the ‘movement-memory nexus’ (Daphi and Zamponi, 2019). This was proposed primarily from the perspective of social movement studies (hence with movement rather than memory as the leading term) and defined as the interplay between *movements about memory*, *memory in movements* and *memories of movements*. The memory-activism and movement-memory nexuses echo each other while reflecting differing priorities and interdisciplinary methodologies that do not always sit easily with each other. Most notable is the difference between the use of the more general term ‘activism’ and the more specific ‘movement’ as keywords. While these terms differ with respect to the continuity, organisation and numbers of actors behind their associated modes of action (see Daphi in this special issue), there is also clearly an overlap between them. In this special issue of *Memory Studies*, we have favoured the idea of a ‘memory-activism nexus’, while also recognising the heuristic value and compatibility of alternatives such as the ‘movement-memory nexus’.

Reflecting priorities in their field, social movement studies scholars have concerned themselves primarily with ‘memory in movements’ (here: ‘memory in activism’) and hence have highlighted the role of memory in shaping the action repertoire of movements and in providing activists with historical reference points (Della Porta et al., 2018; Zamponi, 2013, 2018). Based on a conference held in Utrecht in March 2023, this special issue set out to explore the interactions between all different components of the memory-activism nexus. In doing so, it attempted to better grasp the many factors, processes and actors at work within that nexus by calling on a range of disciplinary, regional and historical expertise while always putting the production and circulation of memory at the centre of its concerns. In their variety, the cases presented here bring into visibility some of the historical, political and cultural variables that affect the operation of the nexus by covering, for example, memory work in conditions of repression (Mousavi), political instability (Fridman), political polarisation (Blom), increased commercialisation (Erbil) and digital surveillance (Merrill), to mention just some of the most salient.

Against the backdrop of this variation, all articles engage specifically, albeit in different ways, with the interplay between memory in activism and the memory of earlier movements, foregrounding in many instances how one can shift into the other. Since memory activism has already received considerable attention, culminating in the recent publication of *The Routledge Handbook of Memory Activism* (Gutman and Wüstenberg, 2023; also reviewed in this issue of *Memory Studies*), the emphasis of this special issue is on other strands of the nexus and hence, in the first instance, on cases where memory is a means to a greater transformative end and not only an end in itself. Resonating with this emphasis, the double meaning of the issue’s title – *Remembering Activism* – is intended to capture what we saw emerging as a continuous feedback loop between activism as the object of remembrance and activism as the agent of new acts of remembrance. In different ways, the articles collected here show the afterlives that previous protests and social movements have in the stories that are later told about them with the help of texts, images, objects and places and, in turn, how the mediated memory of earlier activism informs later activists and movements by feeding back into actions, mobilisations and expectations, though not necessarily in a linear way. The 12 articles, structured here in pairs with shared resonances, reveal the complexity of that feedback, including moments of rupture and discontinuity as well as re-engagement.

The opening essays by Priska Daphi and Orli Fridman provide different perspectives on the sometimes difficult relationship between new and old movements. In ‘Mnemonic adoption and rejection: How activists remember and forget previous mobilisations’, Daphi shows, with reference to the German Global Justice Movement (1998–2007) and the Blockupy movement (2012–2015), how activists’ position-taking in the present entails selectively and strategically remembering predecessors. In ‘Memory and protest in Belgrade: Remembering the 1990s in the mass demonstrations of 2023’, Fridman shows how the memory of earlier protests continues to spill over into later ones but also how that memory, since it is one of defeat, can be inhibiting as much as inspirational.

The next pair of essays both deal with modern Turkey and show, by reference, on the one hand, to a hyper-iconic male activist and, on the other hand, to women activists whose memory has been marginalised, that memorability is context-dependent. In ‘Commodification anxiety and the memory of Turkish Revolutionary Deniz Gezmiş’, Duygu Erbil outlines the gradual reification of the memory of an iconic student leader but also shows how the very availability of this memory, albeit commodified, meant that it could still become a resource for expressing dissent in the Gezi Park protests of 2013. Offering an alternative perspective on the memory of Turkish activism in the 1960s and 1970s, Lucie Drechselová reveals a very different mechanism in ‘Navigating victimhood: Women’s life writing and activist memory in Turkey’. She shows how women, initially excluded from the memory of their political generation, have recently used autobiographical writing to reinscribe themselves in the record and, in doing so, to practice a form of ‘activist victimhood’.

The collected articles also highlight the importance of cultural mediation in the workings of the nexus. While interviews are a key component of some articles, in line with dominant practices in the social sciences, other essays bring to light the role of cultural sedimentation in creating a communicative link between generations that transcends the lifespan of particular movements. In her essay ‘Solidarity: Memory work, periodicals, and the protest lexicon in the long 1960s’, Sophie van den Elzen thus focuses on the role of keywords in creating continuities between protest cycles, showing also how the resignification of old words can be a way of creating new backstories for current-day agendas. Relatedly, Tashina Blom’s ‘My body my choice: The appropriation of feminist cultural memory in American anti-vaccine movements’ looks to political slogans as carriers of memory and shows how their re-use serves to affiliate movements across time but also, in the form of hostile appropriation, to create a disempowering mnemonic rupture with strategic impact in the present.

The next two articles show how embodied performance can become a carrier of memory within new mobilisations, providing links between earlier and later movements. Daniele Salerno’s ‘Walks, marches, parades: LGBT+ activist mnemonic labour in Argentina’ analyses the practice of memory walks as a renewable resource for present-day activism directed towards LGBT+ rights. He shows how the memory walks themselves combine commemorations of past struggle and persecution with celebrations of resilience in the present and how accounts of these performances are then turned into archival records that provide a possible resource for future memory walks. Alexander Thygesen’s ‘Contentious memories of a riot dog: The Matapacos statue intervention during the 2019/2020 Social Uprising in Chile’ shows how, in the context of the Chilean mass mobilisation of 2019, the introduction of a guerrilla statue into the cityscape helped in connecting the protest to long-term struggles within the region and in providing a common point of reference for protesters.

As the historical spread of the various cases reveals, the cultural mediation of activism occurs within changing media ecologies and increasingly within digital platforms whose affordances enable but also constrain memory work. In ‘Dangerous remembering in volatile spaces: Towards activist memory work in the Iranian context’, Nafiseh Mousavi shows how, within conditions of repression at home and of dispersal in the diaspora, digital space has emerged as a prime site for memory; it is there that critics of the regime can engage in acts of remembrance otherwise prohibited in Iran. With reference to very different conditions in the Netherlands, Rik Smit and Sacha van Leeuwen’s ‘Digital activism in the future past: How Extinction Rebellion NL connects past, present and future in their memory work on Instagram’ also shows the importance of the digital media ecology to contemporary activism. They demonstrate how XR activists have been using Instagram as a primary platform for communication but also for recalling earlier protest events as they resonate with new ones.

Samuel Merrill sounds a warning, however, about the implications of digitalisation and datafication for activism in his ‘Remembering like a state: Surveillance databases, digital activist traces, and the repressive potential of mediated prospective memory’. He points to the mass collection of data about protesters on the part of police and intelligence agencies that is currently taking place and warns that these data may be deployed in the future within repressive measures attacking civil liberties. As Merrill’s article shows, not all memory is beneficial to activists such that the memory of activism itself becomes a site of contestation. This point is taken up in Ann Rigney’s ‘Prefigurative remembrance: Archiving as activist mnemonic practice’, which shows with reference to the ‘movements of the squares’ in the period after 2011 how the making and managing of (digital) records has become part of the activist skill set. Rigney presents activist self-archiving as a future-oriented form of memory work aimed at producing a counter-memory that prolongs the impact of protest by giving it a cultural afterlife.

Combined, the articles in this special issue foreground what we and many of the contributing authors refer to as ‘activist memory work’ (Merrill et al., 2020; Till, 2008): this adds a new component to the memory-activism nexus as hitherto defined. Activist memory work refers to the active production of memory as an integral part of activism itself to amplify its impact and hence advance a political cause. As such, it connects all dimensions of the memory-activism nexus and, specifically, expands our understanding of ‘memory in activism’ beyond the instrumentalisation of existing narratives. Activist memory work can play out through the mobilisation and interpretation of existing narratives, the shaping of narratives about ongoing events or the archiving of the present moment to create a future-proof memory that extends the lifespan of a movement and its associated ideals.

In this way, the special issue adds significantly to our understanding of the ‘futural momentum’ (Bryant and Knight, 2019: 134) in the memory-activism nexus. This also gives it a broader relevance within the field of memory studies, which, since at least Gutman et al. (2010), has been increasingly engaging not only with what has come to pass but also with what will come to be. While this prospective orientation is perhaps most explicitly illustrated in the articles written by Smit and Van Leeuwen, Merrill and Rigney, it cuts through the collection as a whole. In multiple ways, the contributors show that remembering is often itself an integral part of collective action directed towards broader transformations. The case studies cover the memory work of activists associated with diverse contemporary and historical causes across the political spectrum (from climate rebels to anti-vaxxers), and with ties to different communities (from diaspora groups to Occupiers) and different generations (from 68 student protestors to those of Gen Z). At the same time, they show that activists in carrying out this work must respond to several other actors whose overall influence within the memory-activism nexus demands further empirical attention. Whether these be police or security agencies, market actors or the algorithms that influence the workings of social media platforms and databases, it is clear that activist memory work operates in a complex field of different forces.

The idea of activist memory work, which for some scholars helps re-politicise the concept of memory work following its widening application to mean all ongoing engagements with the past in the present (Merrill et al., 2020; see also Smit et al., 2018), also encourages researchers to reflect on their own role in the memory-activism nexus. Already in the first issue of *Memory Studies*, Karen Till (2008) called on us as individuals, community members or activists, but also as scholars to ‘consider how memory studies might develop a more socially responsible research practice’ (p. 102). With this in mind, our special issue ends with a provocative intervention by Red Chidgey called ‘Activist turns: The (in)compatibility of scholarship and transformative activism’. In this commentary, Chidgey reflects on the current interest in the intersection between activism and memory and challenges us to draw out its implications for how we conceive of the task of memory scholars within the memory-activism nexus.

Finally, this special issue brings to light a variation of activist memory work that deserves to be taken into consideration as part of the memory-activism nexus but which operates at its fringes and at the threshold between social movements and the defiant actions of individuals. We tentatively call this ‘memory as activism’. As the articles by Drechselova and Mousavi illustrate, remembering can itself constitute a form of low-threshold activism in contexts of repression or marginalisation. Although not necessarily restricted to such contexts, this newly named strand of the memory-activism nexus has the capacity to aid the analysis of scenarios where just to remember something, however briefly and privately, without necessarily the possibility or perhaps even desire to bring about wholesale changes to the mnemonic regime represents a form of resistance and defiance. Memory as activism, in short, captures the sort of activist memory work which stands in contrast not only to the prevailing social and political regimes of its wider setting but also more publicly orientated forms of activist memory work associated with other strands of the nexus.

Closing this editorial on the topic of memory as activism indicates that this special issue is less the wrapping up of a discussion than an invitation to continue the debate. We hope that the readers will continue to empirically and conceptually explore the notion of the memory-activism nexus just as its contributing authors have done. To them, we say thank you for making the issue what it is.
